# The influence of patient’s age on metabolic and bariatric results of laparoscopic sleeve gastrectomy in 2-year observation

**DOI:** 10.1186/s12893-020-00989-0

**Published:** 2020-12-09

**Authors:** Paulina Woźniewska, Inna Diemieszczyk, Dawid Groth, Łukasz Szczerbiński, Barbara Choromańska, Agnieszka Błachnio-Zabielska, Adam Krętowski, Hady Razak Hady

**Affiliations:** 1 1St Department of General and Endocrine Surgery, Medical University of Bialystok, ul. Marii Skłodowskiej-Curie 24A, 15-276 Bialystok, Poland; 2 Eastern Center of the Burns Treatment and Reconstructive Surgery, Independent Public Health Care Center in Łeczna, Łęczna, Poland; 3 Department of Endocrinology, Diabetology and Internal Medicine, Medical University of Bialystok, Bialystok, Poland; 4 Department of Hygiene, Epidemiology and Metabolic Disorders, Medical University of Bialystok, Bialystok, Poland

**Keywords:** Laparoscopic sleeve gastrectomy, Metabolic surgery, Bariatric surgery, Weight loss

## Abstract

**Background:**

The incidence of obesity has been constantly growing and bariatric procedures are considered to be the most effective treatment solution for morbidly obese patients. The results of laparoscopic sleeve gastrectomy (LSG) may differ depending on patient’s age, gender, preoperative body mass index (BMI) and physical activity.

**Methods:**

The aim of this study was to evaluate age-related differences in the outcome of LSG in terms of weight loss parameters, lipid and carbohydrate profile. The retrospective analysis of 555 patients who had undergone LSG was performed to compare the metabolic outcomes of surgery in individuals < 45 and ≥ 45 years old. Evaluation of weight loss parameters along with selected laboratory data was performed to demonstrate the results of LSG in 2 years follow-up.

**Results:**

Overall, 238 males and 317 females (43%/57%) with median age of 43 years and median preoperative BMI of 46.41 (42.06–51.02) kg/m^2^ were analyzed. Patients in both groups presented significant weight loss at 24 months after the surgery with comparable percentage of total weight loss (40.95% in < 45 years old group and 40.44% in ≥ 45 years old group). The percentage of excess weight loss (78.52% vs. 74.53%) and percentage of excess BMI loss (91.95% vs. 88.01%) were higher in patients < 45 years old. However, the differences were not statistically significant (*p* = 0.662, *p* = 0.788 respectively). Patients under 45 years old experienced faster decrease in fasting glucose level that was observed after only 3 months (109 mg/dl to 95 mg/dl in < 45 years old group vs. 103.5 mg/dl to 99.5 mg/dl in ≥ 45 years old group, *p* < 0.001). Both groups presented improvement of lipid parameters during the observation. However, patients < 45 years old achieved lower values of LDL at 3 and 12 months follow-up (115 mg/dl vs. 126 mg/dl, *p* = 0.010; 114.8 mg/dl vs. 122 mg/dl, *p* = 0.002). Younger group of patients also showed superior improvement of triglycerides level.

**Conclusions:**

LSG results in significant weight loss in all patients regardless age. In turn, superior and faster improvement in lipid and carbohydrate profile is achieved in patients under 45 years old.

## Background

The increase in prevalence of overweight and obesity has become a major health care challenge for many countries. The recent data show that there are 107.7 and 603.7 of obese respectively between children and adults, what proves that obesity pandemic affects all age groups [1]. However, obesity and ageing are essential risk factors for cardiovascular diseases and premature mortality [2, 3]. These facts emphasize the need for constant focus on evidence-based intervention to prevent and effectively treat obese patients. In many cases, conservative methods such as dietary changes, increased physical activity and drugs therapy are not sufficient to significantly reduce excessive body mass. As a result, metabolic and bariatric surgery has been gaining unquestionable popularity as a method of obesity treatment. Bariatric procedures not only reduce the number of morbidly obese patients, but also significantly improve the quality of patients’ lives [4]. Among all surgical procedures, laparoscopic sleeve gastrectomy (LSG) plays a significant role as a separated method due to satisfactory weight loss, resolution of comorbidities and safety of performance [5–7]. Nowadays, laparoscopic sleeve gastrectomy is the most commonly performed bariatric procedure in the United States that shows the evident increase in the frequency of performance from 3% in 2008 to 54% in 2014 in comparison with laparoscopic adjustable gastric banding (LAGB) and Roux-en-Y gastric bypass (RYGB) [8]. The metabolic effect of LSG has been widely analyzed in both short and long-term studies. However, there is still a deficiency of research that investigate factors affecting the weight loss and metabolic parameters in patients undergoing LSG. The predictors of satisfactory results after LSG include age, gender, preoperative weight loss, extra physical activity and different modifications of operating technique [9, 10]. A few reported studies suggest that younger patients may achieve more satisfactory weight loss results. However, these studies were carried out for a shorter follow-up, and as it is known, long-term observation is crucial to analyze the effects of bariatric procedures [9, 11].

## Methods

This is a retrospective cohort study of consecutive patients undergoing laparoscopic sleeve gastrectomy between 2014 and 2016 at University Hospital in Poland. All procedures were performed by an expert surgeon. Patients were qualified to surgical treatment of morbid obesity according to the European Guidelines on Metabolic and Bariatric Surgery [12]. The inclusion criteria for the surgical procedure comprised of inability to achieve sustained weight loss with conservative management, BMI ≥ 40.0 kg/m^2^ or 35–40 kg/m^2^ with the presence of at least one obesity-related co-morbidity such as type 2 diabetes, hypertension, obstructive sleep apnea, non-alcoholic fatty liver disease (NAFLD) and steatohepatitis (NASH), hyperlipidemia and others. Patients with obesity-related endocrine diseases, clinically significant or unstable mental health concerns, addicted to alcohol or psychostimulants and women planning on pregnancy within 2 years after a potential surgery were excluded from the bariatric procedure. Study was designed and conducted according with STROBE guidelines and the report of the International Society for Pharmacoeconomics and Outcomes Research (ISPOR) task force [13, 14].

Patients ≥ 45 years old were compared with a group of patients < 45 years. Patients were excluded, when lack of necessary data.

The study was approved by the Bioethics Committee of the Medical University of Bialystok (the reference number of the consent: R-I-002/248/2018).

### Data collection

Demographic and clinical data were gathered prospectively along with repetitive measurements of selected laboratory parameters including fasting glucose and insulin concentration, glycated hemoglobin level (HbA1c), C-reactive protein (CRP), total cholesterol and its fractions, triglycerides (TG), alanine aminotransferase (ALT) and aspartate aminotransferase (AST). All parameters were examined on the day of operation, 1 month, 3 months, 6 months, 12 months and 24 months after LSG. Bariatric effects were assessed using following equations:

- Percentage of total weight loss (%TWL): ${\text{\% TWL }} = { }\frac{{{\text{initial weight}} - {\text{current weight}}}}{{\text{initial weight}}}{ } \times 100$
- Percentage of excess BMI loss (%EBMIL): ${\text{\% EBMIL }} = { }\frac{{{\text{initial BMI}} - {\text{postoperative BMI}}}}{{{\text{initial BMI}} - 25}}{ } \times 100{ }$
- Percentage of excess weight loss (%EWL): ${\text{\% EWL }} = { }\frac{{{\text{initial weight}} - {\text{postoperative weight}}}}{{{\text{initial weight}} - {\text{ideal weight}}}}{ } \times 100$, while an ideal weight is defined by the weight corresponding to the BMI of 25 kg/m^2^

Homeostatic model assessment for insulin resistance index (HOMA-IR) was calculated to determine insulin resistance (IR) in patients. Values > 2.5 were defined as IR.

### Surgical technique

All patients underwent laparoscopic sleeve gastrectomy performed by the same surgeon and 2 alternating assistants. The procedure started with the formation of pneumoperitoneum by the use of CO_2_ introduced to the peritoneal cavity by the Veress needle. The gas was insufflated until the intraabdominal pressure of 12–15 mmHg. The dissection of greater gastric curvature started approximately 6 cm proximally to the pylorus and was continued to the angle of His. The reduction in stomach volume was performed using a 36-Fr bougie and linear staplers. At the end, the leak test was performed with the use of air and 5% glucose solution. The resected stomach specimen was subjected to a histopathological examination.

Patients were discharged home on the second day after the surgery if no complications had occurred. The oral fluids were given on the day after the operation. Patients were kept on liquid diet for at least two weeks. Then, solid aliments were gradually introduced within 30 days.

### Statistics

Data was analyzed using Statistica 13.5 software (StatSoft®, Tulsa, Oklahoma, United States of America). Continuous values were presented as means with standard deviations, or medians with interquartile ranges as appropriate. Quantitative variables were compared using t Friedman’s ANOVA with multiple comparison of ranges sum (nonparametric). p-values ≤ 0.05 were considered statistically significant.

## Results

The study group included 555 patients—238 males and 317 females (43%/57%), in median age of 43 (36–53) years. Median preoperative BMI was 46.41 (42.06–51.02) kg/m^2^.

There were 254 patients in ≥ 45 years group (46%), and 301 in < 45 years group (54%). Sex distribution did not differ between groups significantly (*p* = 0.233). In the group of patients aged ≥ 45 years old there were 102 males and 152 females (40%/60%), while in the group under 45 years old patients there were 136 males and 165 females (45%/55%). Median BMI on the day of operation was 46.69 (42.02–52.08) kg/m^2^ in ≥ 45 years old group and 46.25 (42.17–50.31) kg/m^2^ in < 45 years old group and did not differ significantly between age groups (*p* = 0.410). Follow-up rates at 1, 3, 6, 12 and 24 months were 88.3% (490/555), 80.7% (448/555), 70.5% (391/555), 64.1% (356/555) and 45.2% (251/555) respectively and were comparable between two age groups. Overall, 35.7% of participants had hypertension. Type 2 diabetes was present in 16.8% and insulin resistance in 14.9% of patients. Mean type 2 diabetes mellitus duration was 7.52 (3.88–14.16) years in ≥ 45 years old group and 3.42 (0.52–12.89) years < 45 years old. Among 53 patients over 45 years old with type 2 diabetes mellitus, 35 needed insulin treatment (66%), while 18 patients were on oral antidiabetic therapy (34%). It was respectively 11 vs. 29 patients (27.5% vs. 72.5%) in < 45 years old group.

Prevalence of comorbidities was higher in patients ≥ 45 years old. Table 1 present results of repetitive measurements of selected laboratory parameters in general population during the 2 years observation, while Table 2 demonstrates comparison of selected laboratory parameters and comorbidities between two age groups before the surgical procedure (Tables 1, 2).

**Table 1:** Repetitive measurements of selected laboratory parameters in all patients during the follow-up

Variables	0	1 month	3 months	6 months	12 months	24 months	p-value
CRP (mg/l)	6.81 (3.7–11.1)	5.28 (2.8–10.1)	5.7 (2.6–10.1)	5.91 (1.98–13.85)	2.4 (0.8–6.2)	5.2 (1–8-9.3)	< 0.001
FPG (mg/dl)	106 (98–122)	101 (92–111.5)	98 (89–105)	98 (90–108)	93 (87–100)	93 (87–101)	< 0.001
Insulin (mIU/l)	22.1 (14.6–32.35)	11.8 (7.95–16.3)	10.65 (7.4–14.8)	8.5 (5.3–15.1)	8.3 (6.1–11.9)	12.8 (8–17.4)	< 0.001
HOMA-IR	5.85 (3.61–9.81)	2.74 (1.79–3.81)	2.31 (1.51–3.32)	1.78 (1.07–3.47)	1.65 (1.21–2.53)	2.63 (1.7–3.78)	< 0.001
ALT (IU/l)	33 (22.95–47)	35 (24.5–47.1)	24 (18–32.8)	19.2 (14–27)	20.25 (15.8–27)	19 (14–23)	< 0.001
AST (IU/l)	26 (19.25–38.6)	27 (21–36)	22 (17–28.3)	19 (15.2–26)	20.55 (16.1–25)	19 (16–23.8)	< 0.001
Cholesterol (mg/dl)	204.00 (176–230)	174 (154–198)	178 (157–200)	196 (173–222)	179 (163–205)	187 (166–209)	< 0.001
LDL (mg/dl)	138.00 (113–116)	116 (97–141)	120 (98–141)	131 (106.1–154)	118.25 (99–138.7)	115 (98–131)	< 0.001
TG (mg/dl)	156 (112–198)	133 (104–171)	123 (96–151)	49 (39–60)	93 (73–126)	122 (93–143)	< 0.001
HDL (mg/dl)	46 (38–58)	37 (31–44)	43 (36–49)	49 (39–60)	55 (47–64)	53 (47–63)	< 0.001

Follow-up rates at 1, 3, 6, 12 and 24 months were 88.3% (490/555), 80.7% (448/555), 70.5% (391/555), 64.1% (356/555) and 45.2% (251/555) respectively

Values are expressed as median [IQR]

CRP, C-reactive protein; FPG, fasting plasma glucose; HOMA-IR, homeostatic model assessment of insulin resistance index; ALT, alanine transaminase; AST, aspartate transaminase; LDL, low-density lipoprotein; TG, triglyceride; HDL, high-density lipoprotein; HbA_1c_, glycated hemoglobin

**Table 2:** Repetitive measurements of selected laboratory parameters and comparison of comorbidities in two age group

Variables	< 45 (n = 301)	≥ 45 (n = 254)	p-value
Female/male	165/136 (55/45%)	152/102 (60%/40%)	0.453
BMI (kg/m^2^)	46.25 (42.17–50.31)	46.69 (42.02–52.08)	0.410
FPG (mg/dl)	109 (100–132)	103.5 (94.5–113)	< *0.001*
Insulin (mIU/l)	20.6 (13.6–29.3)	24.3 (16.5–34.5)	*0.003*
HOMA-IR	5.41 (3.26–8.78)	6.8 (4.19–10.29)	< *0.001*
HbA_1c_ (%)	5.5 (5.2–5.9)	5.8 (5.4–6.6)	< *0.001*
Cholesterol (mg/dl)	199 (172–228)	203.5 (181–236)	0.013
LDL (mg/dl)	133.2 (113–158)	141.7 (112–174.7)	0.069
HDL (mg/dl)	45 (38–56)	47 (39–59.5)	0.115
TG (mg/dl)	148 (107–191.5)	159.5 (121–205)	*0.022*
Hypertension	83 (27.6%)	115 (45.2%)	0.211
Type 2 diabetes mellitus	40 (13.3%)	53 (20.9%)	0.115
Insulin resistance	38 (12.6%)	45 (17.7%)	*0.022*
Dyslipidemia	64 (21.3%)	70 (27.6%)	0.115

BMI, body mass index; FPG, fasting plasma glucose; HbA_1c_, glycated hemoglobin; HDL, high-density lipoprotein; HOMA-IR, homeostatic model assessment of insulin resistance index; LDL, low-density lipoprotein; TG, triglyceride

### Weight loss results (Table 3)

**Table 3:** Repetitive measures of bariatric effects in all patients

Variables	0	1 month	3 months	6 months	12 months	24 months	p-value
BMI (kg/m^2^)	46.41 (42.06–51.02)	41.18 (37.24 (46.11)	36.98 (32.9–42.81)	33.31 (29.41–38.98)	30.84 (26.49–36.93)	27.16 (23.5–33.46)	< 0.001
%EWL	N/A	19.20 (15.30–23.76)	36.25 (28.06–44.11)	51.38 (40.58–60.87)	62.24 (48.63–75.68)	76.92 (62.27–90.91)	< 0.001
%EBMIL	N/A	22.65 (17.61–28.20)	42.16 (32.4–52.71)	59.71 (45.64–74.24)	71.74 (54.55–90.69)	71.74 (69.87–108.36)	< 0.001

BMI, body mass index; %EBMIL, percentage of excess BMI loss; %EWL, percentage of excess weight loss

The %TWL, %EWL and %EBMIL showed statistically significant increase during the follow-up in both groups (Table 3). However, the results did not reach statistical significance when compared between groups. Mean %TWL after two years of observation was comparable between groups and reached 40.95% in < 45 years old group and 40.44% in ≥ 45 years old group (*p* = 0.459). In ≥ 45 years old group %EWL at 24 months after surgery was 74.54%, while in < 45 years old group it was 78.52% (*p* = 0.622). Mean %EBMIL in ≥ 45 years old group was 88.01% at 2 years follow-up, while in < 45 years old group it was 91.95% (*p* = 0.788, Table 4).

**Table 4:** Repetitive measures of weight loss effect after LSG by age groups

Variables		0	1 month	3 months	6 months	12 months	24 months	p-value
BMI (kg/m^2^)	< 45	46.25 (42.17–50.31)	40.81 (37.32–45.73)	36.57 (32.88–41.81)	33.21 (29.32–38.76)	30.38 (26.42–35.82)	26.77 (23.78–32.25)	< 0.001
BMI (kg/m^2^)	≥ 45	46.69 (42.02–52.08)	41.36 (37.11–47.27)	37.62 (33.03–44.25)	33.83 (29.75–39.68)	31.83 (26.77–37.65)	27.62 (23.18–34.72)	< 0.001
p-value ≥ 45 vs. < 45		0.410	0.501	0.383	0.405	0.187	0.596	
%TWL	< 45	N/A	10.14 (8.16–12.26)	19.37 (16.20–22.27)	27.01 (22.22–31.19)	33.33 (26.97–38.38)	40.95 (35.26–45.97)	< 0.001
%TWL	≥ 45	N/A	10.23 (8.15–11.82)	18.33 (15.65–22.00)	26.52 (22.53–30.47)	32.47 (27.33–38.03)	40.44 (35.95–45.10)	< 0.001
p-value ≥ 45 vs. < 45		N/A	0.581	0.288	0.551	0.979	0.459	
%EWL	< 45	N/A	19.24 (15.38–24.53)	36.86 (29.24–44.20)	51.67 (40.98–61.54)	63.69 (48.57–75.86)	78.52 (62.65–90.32)	< 0.001
%EWL	≥ 45	N/A	19.18 (15.3–22.95)	35.29 (27.56–43.85)	50 (40.41–59.59)	60 (48.68–73.77)	74.54 (61.43–90.91)	< 0.001
p-value ≥ 45 vs. < 45		N/A	0.499	0.361	0.493	0.412	0.622	
%EBMIL	< 45	N/A	22.8 (17.61–28.89)	43.26 (33.42–53.35)	61.24 (46.02–74.45)	74.26 (56.04–90.79)	91.95 (70.74–107.16)	< 0.001
%EBMIL	≥ 45	N/A	22.3 (17.39–27.31)	41.11 (31.53–51.45)	58.58 (44.81–72.93)	69.66 (54.38–89.92)	88.01 (68.7–111.25)	< 0.001
p-value ≥ 45 vs. < 45		N/A	0.486	0.354	0.463	0.376	0.788	

BMI, body mass index; %EBMIL, percentage of excess BMI loss; %EWL, percentage of excess weight loss; %TWL, percentage of total weight loss

### Carbohydrate parameters

Fasting glucose level decreased significantly during the follow-up in both groups (*p* < 0.001), but to a greater extent in < 45 years old group at every observation period (*p* = 0.001 at 12 months and *p* = 0.005 at 24 months). Patients < 45 years old experienced a faster decrease in glucose level that was observed after only 3 months (109 mg/dl to 95 mg/dl vs. 103.5 mg/dl to 99.5 mg/dl, *p* < 0.001). The glycated hemoglobin level differed the groups significantly in favor of < 45 years old patients at every point of the study with the exception of 24-month measurements (*p* = 0.064). The decrease in mean insulin concentration was also noticed during the follow-up in both groups (*p* < 0.001), however the measurements did not differ groups significantly. The same pattern was observed for HOMA-IR, which showed statistically significant decrease during observation period (*p* < 0.001). Nonetheless, there was no statistically significant differences between the groups in 24-month follow-up (Table 5).

**Table 5:** Repetitive measurements of carbohydrate profile after LSG by age groups

Variables		0	1 month	3 months	6 months	12 months	24 months	p-value
FPG (mg/dl)	< 45	109 (100–132)	98 (90–108)	95 (87–102)	97 (89–106)	92 (84–98)	92 (87–98)	< 0.001
FPG (mg/dl)	≥ 45	103.5 (94.5–113)	105 (95–115)	99.5 (91–108)	100 (92–109)	95 (90–100)	97 (88–103)	< 0.001
p-value ≥ 45 vs. < 45		< *0.001*	< *0.001*	< *0.001*	*0.026*	*0.001*	*0.005*	
Insulin (mIU/l)	< 45	20.6 (13.6–29.3)	11.5 (7.9–16.7)	10.15 (7.2–14.5)	9.2 (5.1–15.7)	7.9 (6–12.5)	11.9 (7.9–18.7)	< 0.001
Insulin (mIU/l)	≥ 45	24.3 (16.5–34.5)	11.8 (8.1–16.1)	10.85 (7.8–15.6)	8.3 (5.5–13.7)	8.6 (6.8–11.8)	13.3 (8.2–16.75)	< 0.001
p-value ≥ 45 vs. < 45		*0.003*	0.227	0.281	0.978	0.240	0.784	
HOMA-IR	< 45	5.41 (3.26–8.78)	2.69 (1.63–3.71)	2.22 (1.42–3.26)	1.84 (0.97–3.66)	1.50 (1.14–2.64)	2.59 (1.60–3.77)	< 0.001
HOMA-IR	≥ 45	6.8 (4.19–10.29)	2.86 (1.86–4.09)	2.53 (1.63–3.39)	1.75 (1.17–3.03)	1.77 (1.36–2.49)	2.81 (1.71–3.76)	< 0.001
p-value ≥ 45 vs. < 45		< *0.001*	0.060	0.060	0.748	0.104	0.886	
HbA_1c_ (%)	< 45	5.5 (5.2–5.9)	5.3 (5–5.8)	5.2 (5–5.6)	5.2 (4.7–5.4)	5.1 (5–5.4)	5.4 (5.1–5.6)	< 0.001
HbA_1c_ (%)	≥ 45	5.8 (5.4–6.6)	5.7 (5.3–6.2)	5.4 (5.2–5.9)	5.4 (4.9–5.7)	5.3 (5.1–5.6)	5.5 (5.1–5.8)	< 0.001
p-value ≥ 45 vs. < 45		< *0.001*	< *0.001*	< *0.001*	*0.001*	*0.001*	0.064	

FPG, fasting plasma glucose; HOMA-IR, homeostatic model assessment of insulin resistance index; HbA_1c_, glycated hemoglobin

The superior improvement of glucose metabolism in patients < 45 years old could be probably associated with lower percentage of type 2 diabetes cases and shorter duration of disease.

### Lipid parameters

Patients < 45 years old demonstrated significantly lower total cholesterol values at 1, 3, 6 and 12 months (*p* = 0.009, < 0.001, 0.007, 0.001 respectively) when compared to ≥ 45 years old group. However, at 24 months the groups did not differ significantly in terms of mean total cholesterol level. The mean triglycerides level decreased consistently in both groups during the follow-up (*p* < 0.001). Nonetheless, patients < 45 years old demonstrated lower mean values of triglycerides at 1, 3 and 12 months (*p* = 0.004, 0.002, < 0.001 in sequence). Both groups also showed a significant decrease in LDL level during the 24-month observation (*p* < 0.001). Comparison between groups revealed statistically significant differences at 3 (*p* = 0.010) and 12 months (*p* = 0.002) in favor of < 45 years old group. The statistically significant increase in mean HDL cholesterol level was seen during the follow-up for both groups (*p* < 0.001). Patients < 45 years old reported better results in mean level of HDL at 1 (*p* = 0.016) and 6 months (*p* = 0.010, Table 6).

**Table 6:** Repetitive measurements of lipid profile after LSG by age groups

Variables		0	1 month	3 months	6 months	12 months	24 months	p-value
Cholesterol (mg/dl)	< 45	199 (172–228)	170 (151–191)	173 (156–198)	189 (173–212)	175 (156–198)	182 (164–204)	< 0.001
Cholesterol (mg/dl)	≥ 45	203.5 (181–236)	178 (156–205)	186.5 (162.5–214)	205 (174–229)	188.5 (165–213.5)	190 (168–212)	< 0.001
p-value ≥ 45 vs. < 45		*0.013*	*0.009*	< *0.001*	*0.007*	*0.001*	0.082	
LDL (mg/dl)	< 45	133.2 (113–158)	115 (96–136)	115 (95–136)	127 (106.1–151)	114.8 (94–129)	114 (97–130)	< 0.001
LDL (mg/dl)	≥ 45	141.7 (112–174.7)	120 (99–143)	126 (100–145.6)	135 (105.1–158)	122 (103.9–148.5)	116 (100–132	< 0.001
p-value ≥ 45 vs. < 45		0.069	0.219	*0.010*	0.230	*0.002*	0.303	
TG (mg/dl)	< 45	148 (107–191.5)	127 (99–167)	114 (91–147)	109 (79–149)	86 (68–118)	120 (87–140)	< 0.001
TG (mg/dl)	≥ 45	159.5 (121–205)	142.5 (109–176)	129.5 (101–156)	106 (80–143)	101 (78–134)	123 (100–147)	< 0.001
p-value ≥ 45 vs. < 45		*0.022*	*0.004*	*0.002*	0.931	< *0.001*	0.114	
HDL (mg/dl)	< 45	45 (38–56)	36 (31–42)	43 (36–49)	48 (37–59)	54 (47–64)	54 (47–62)	< 0.001
HDL (mg/dl)	≥ 45	47 (39–59.5)	38 (32–45)	44 (37–49.5)	51 (42–62)	56 (47–63)	52 (46–63)	< 0.001
p-value ≥ 45 vs. < 45		0.115	*0.016*	0.384	*0.010*	0.509	0.771	

LDL, low-density lipoprotein; TG, triglyceride; HDL, high-density lipoprotein

## Discussion

The incidence of obesity and its comorbidities has been constantly increasing, therefore the prevalence of bariatric procedures continues to rise accordingly [15]. Among all surgical approaches, laparoscopic sleeve gastrectomy stands out as being of crucial importance. It has been widely proven that LSG results in significant weight loss and resolution of obesity related diseases [16–18]. The comparison of two groups in our study (< 45 vs. ≥ 45 years old patients) reveals that all patients regardless the age, benefit from the surgical procedure in terms of considerable body mass reduction and improvement of lipid and carbohydrate metabolism. However, patients < 45 years old tend to have better and faster results when compared with group ≥ 45 years old.

Patient’s age is a crucial determinant for many medical interventions, including the results of bariatric procedures. Differences in energy requirements, psychological condition as well as physiopathological and behavioral hypothesis may explain the association between age and weight loss after bariatric treatment. Toth et al. reported that aging is associated with a lower lipolytic capacity, what can explain increased adipose tissue accumulation in older patients [19, 20]. Additionally, total energy expenditure decreases with age, which is mainly associated with reduction in physical activity [21, 22]. Younger subjects lead more active lifestyle and have better exercises tolerance, which may also contribute to greater weight loss after bariatric procedure [23]. Social influence is another important factor for weight loss. Studies demonstrate that social issues play major role as a motivating factor for younger patients rather than older ones [24].

Aslaner et al. conducted the research comparing patients under and over 40 years old and observed that the %EBMIL in 1 year follow-up was 82.95 ± 21.88% and 56.75 ± 15.90%, respectively. However, only 55 patients were included in their study [25]. Increase in %EBMIL was also revealed in the study carried out by Contreras et al. They observed statistically significant differences in %EBMIL 12 months after the bariatric procedure (LSG either Roux-en-Y gastric bypass) in favor of patients < 45 years old. Their analysis revealed that only five patients (2.6%) had lost less than 50% EBMIL in the group < 45 years old. Whereas, in the group ≥ 45 years old this percentage was 14.9% (21 patients) [26]. Recent research conducted by Angrisini et al. determined at five-year observation that younger age at the day of surgery is associated with a superior results in %TWL (*p* < 0.001) [27]. On the other hand, research conducted by Gonzalez-Heredia et al. did not reveal any significant relationship between age and %EWL at 6, 12, and 24 months postoperatively [28]. The present study also did not show significant differences in %EWL and %EBMIL in patients across age groups during the observation period.

Improvement of lipid and carbohydrate profile after bariatric surgery in our study is in line with other researches [29, 30]. Amelioration of carbohydrate metabolism after LSG occurs not only because of the body mass reduction but also due to the neurohormonal changes in digestive tract, gut microbiota and bile acids. Reduction in stomach volume results in removal of cells producing ghrelin, which are mainly located in fundus. Decrease in ghrelin concentration leads to appetite reduction and drop in glucose concentration. In consequence, insulin production increases and the improvement of insulin resistance is observed [31]. The “hindgut” hypothesis states that normalization of glucose concentration after bariatric surgery is associated with accelerated nutrients delivery to the distal gastrointestinal tract, that leads to the increased secretion of glucagon-like peptide 1 (GLP-1) and peptide YY (PYY) [32, 33]. Increased secretion of GLP-1 stimulates insulin secretion and inhibits glucagon liberation contributing to the limitation of postprandial glucose excursions. L cells located in the distal part of small bowel produce PYY, that affects the hypothalamus (arcuate nucleus) to signal satiety [34]. The improvement of glucose metabolism after bariatric surgery is also associated with alternations in the levels and types of bile acids that are involved in the enterohepatic circulation. Bile acids facilitate the secretion of fibroblast growth factor 19 and 21 (FGF 19 and 21) in the gut, that circulate to the liver and activate the membrane G protein-coupled receptor (TGR 5). Activation of TGR 5 stimulates the liberation of PYY, GLP-1 and GLP-2 [35, 36]. In the present study decrease in glucose concentration was noticed during the observation in favor of patients < 45 years old. The ABCD grading system and DiaRem score were elaborated in order to predict the success of bariatric surgery in relation to type 2 diabetes mellitus and amelioration of carbohydrate profile. In both scales, advanced age is associated with higher score which is related to minor effect of surgical procedure [37]. Study conducted by Wool et al. comparing obese males ≥ 60 (group II) and 50–59 (group I) years old showed that diabetes improvement was observed in 87% of patients in group I and 90% of patients in group II [38]. Superior results in terms of carbohydrate profile in patients < 45 years old may be due to a lower incidence of impaired glucose tolerance and type 2 diabetes in comparison with patients ≥ 45 years old.

Additionally, duration of diabetes mellitus and preoperative insulin treatment are strong predictors of achieving complete remission after the surgery. There is an evidence that longer duration of diabetes and need for insulin supplementation prior to the surgery correlate negatively with the chance for complete diabetes remission after the bariatric procedure [39]. Patients with longer duration of type 2 diabetes mellitus have reduced beta-cell function and impaired secretory capacity. This results in need for insulin preoperatively and limited success of disease remission after the bariatric surgery [40, 41].

Obesity and components of the metabolic syndrome are the major risk factors for cardiovascular morbidity and mortality. Improvement of lipid parameters reduces the risk of cardiovascular diseases [42, 43]. In our study, the improvement in mean values of total cholesterol, LDL, HDL and triglycerides was noticed during the observation. Although all patients presented better results in lipid parameters after LSG, younger objects (< 45 years old) achieved it faster. The statistical differences between groups were observed especially for total cholesterol analysis in favor of < 45 years old group. Our findings on improving lipid parameters are consistent with those available in the literature [44, 45].

Our results could have been more robust if patients had been matched in terms of preoperative comorbidities including hyperlipidemia, type 2 diabetes mellitus and hypertension. Group bias may interfere with the evidence accuracy. Additionally, longer observation with higher follow-up rate should be performed in order to achieve strong evidence that patient’s age is related to the effect of LSG.

## Conclusions

This study supports the evidence that laparoscopic sleeve gastrectomy is an effective method for substantial and sustainable weight loss in patients with morbid obesity regardless their age. We also demonstrate that lipid and carbohydrate balance improved during the 2-year observation in favor of patients under 45 years old. While above results are not strong enough to firmly support the thesis that lower age is a predicting factor for better and faster amelioration in metabolic profile, our study highlights the necessity of more research into the factors affecting the outcome of LSG.

## Data Availability

All data are available from the corresponding author upon reasonable request.

## Abbreviations

ALT: Alanine aminotransferase
AST: Aspartate aminotransferase
BMI: Body mass index
CRP: C-reactive protein
%EBMIL: Percentage of excess BMI loss
%EWL: Percentage of excess weight loss
FGF 19: Fibroblast growth factor 19
FGF 21: Fibroblast growth factor 21
FPG: Fasting plasma glucose
GLP-1: Glucagon-like peptide 1
HbA1c: Glycated hemoglobin level
HDL: High-density lipoprotein
HOMA-IR: Homeostatic model assessment for insulin resistance index
IR: Insulin resistance
ISPOR: The International Society for Pharmacoeconomics and Outcomes Research
LAGB: Laparoscopic adjustable gastric banding
LDL: Low-density lipoprotein
LSG: Laparoscopic sleeve gastrectomy
N/A: Not applicable
NAFLD: Non-alcoholic fatty liver disease
NASH: Non-alcoholic steatohepatitis
PYY: Peptide YY
RYGB: Roux-en-Y gastric bypass
TG: Triglycerides
TGR 5: Membrane G protein-coupled receptor
%TWL: Percentage of total weight loss
